# M. Hubert Rodrigues on the General Paralysis of the Insane

**Published:** 1848-07-01

**Authors:** 


// THE JOURNAL

OF

PSYCHOLOGICAL MEDICINE

AND

MENTAL PATHOLOGY.

JULY 1, 1848.

### Analytical Reviews.

ART. I.—*Traité de la Paralysie Génêral Chronique, considerê speciale-ment chez les Aliênês.* Par HUBERT RODRIGUES, Professeur Agregé à la Faculté de Montpelier, &c. Anvers, 1847.

*A Treatise on Chronic General Paralysis, considered especially as affecting the Insane.* By HUBERT RODRIGUES, Professor of Medicine at Montpelier, &c. Antwerp, 1847. 8vo, pp. 250.

IT is a remarkable fact, that no monograph on this highly interesting subject has been published in this country. We are principally indebted to French authors for our information on that peculiar form of paralysis which affects the insane. The first *notice* of the affection is to be found in a work of M. Bayle's, which appeared in 1822, entitled, "*Recherches sur les Maladies Mentales.*" The first *distinct treatise,* however, was published by M. Delaye, in 1822, and was styled—"Quelques Considérations sur une espèce de Paralysie qui affecte particulièrement les Aliénés." Since then, Messrs. Bayle and Calmeil have written most ably on the subject. The work before us is the most recent which has issued from the press, and is a systematic and comprehensive treatise.

M. Rodrigues appears to have had extensive opportunities for observation at the various lunatic asylums in France, and his views seem deserving of attention from the circumstances of his having obtained a prize from the Chirurgical Society of Emulation at Montpelier, for the best essay on the General Paralysis of the Insane. This essay was published in the "Revue Medicale," t. ii., 1838.

In the systematic work before us, M. Rodrigues has industriously collected all that is known respecting the singular malady of which it treats. He is, however, not merely a compiler. He has brought forward some original views, and given the details of several interesting pathological alterations, which demand especial notice.

The treatise is divided into three parts. The first two are devoted to the detail of cases. The third relates to the history of the disease, and comprises an account of the predisposing causes, symptoms, state of the mind, duration, complications, prognosis, diagnosis, pathological alterations, &c. We shall commence our observations with the third part, referring incidentally, as occasion may require, to the cases contained in the other portion of the work.

The first points on which we shall dwell at some length, are those relating to predisposing causes, and the condition of the system most conducive to general paralysis. Predisposing causes are those which have reference to the state of the constitution, the action of the external world, and the influence of some particular disease. M. Calmeil and other writers have come to the conclusion that men are more liable to the disease than women. M. Rodrigues is not only of this opinion, but he also considers that men are more predisposed to every kind of cerebral affection than women, in consequence of their proneness to sensuality, their greater intellectual exertions, and their professional anxieties and disappointments. He also considers that men are particularly liable to disease of the brain when an inactive life succeeds years of incessant toil. This opinion is plausible, but it is not borne out by the experience of the majority of writers on insanity. All writers are agreed, however, that men are more subject to paralysis than women.

The predisposition to general paralysis varies with age. It is rarely seen before twenty-five, is less rare from twenty-five to thirty, increases in frequency up to sixty, and then diminishes. M. Bayle has never met with it in infants, nor between fifteen and twenty years of age. M. Calmeil has only witnessed it twice before thirty-two. M. Rodrigues says he has seen it occur in three cases before the age of fifteen. But these instances can hardly be said to affect the general rule laid down by Bayle and Calmeil. They can scarcely be termed exceptions, inasmuch as two of the patients were idiots, and the other was a child three years of age, who had been subject to convulsions. These cases cannot, therefore, be regarded as fair examples of the peculiar form of paralysis under consideration; as this has distinct and remarkable symptoms dependent on a state of brain which it is supposed cannot be developed in idiots and very young children.

M. Bayle noticed that general paralysis of the insane commences with ambitious monomania, and M. Rodrigues has ingeniously suggested that this may arise from the circumstance of the disease generally appearing at that time of life when the passion of ambition is strongest.

The disease attacks individuals of every kind of temperament. The sanguineous and robust appear, however, to be most liable to the complaint. Some of the cases which we lately witnessed have occurred in particularly robust artisans. Profession has some influence in causing the affection. Military men appear to be peculiarly liable to it. Many of Napoleon's soldiers, during the retreat from Moscow, are said to have been affected with this form of general paralysis, owing, no doubt, in a great measure to their mental and bodily sufferings and their intemperance. M. Rodrigues thinks that the proud disposition of the soldier subjects him to the malady. Next to military men, revenue officers seem to be most predisposed to it. Calmeil says that four out of five cases in his practice occurred in this class, and he considers their exposure to cold and damp air at night as a cause of their liability to the disease. M. Rodrigues states that miners are liable to trembling limbs, the first stage of paralysis and premature old age. The peculiar organization which constitutes hereditary taint is no doubt a powerfully predisposing cause. Venereal excesses, intemperance, excessive intellectual labour, syphilitic disease, abuse of mercury, deranged catamenia, have doubtless each an influence in deranging the nervous system, and thus rendering it liable to paralytic disease.

It has been generally stated that this complaint is rare in southern latitudes. Dr. Vulques, physician to the Asylum at Aversa, in Naples, informed Esquirol that amongst 500 insane patients of both sexes, he had only two or three paralytic cases. M. Rodrigues, however, gives some statistics which show that the disease is more prevalent at Lisbon than in France. Dr. Burrows thought that the disease was much more prevalent in France than in England. But since the complaint has been more generally known, it is to be recognised in all the English institutions; and by the reports of the asylums in Scotland and Ireland, which have been lately published, we perceive that the malady is not uncommon in the northern parts of the British isles. These statements render it very doubtful whether climate has much influence in causing a predisposition to the complaint.

An apoplectic habit of body and hypertrophy of the heart must also be enumerated amongst the predisposing causes. M. Rodrigues considers that when the obstacle which produces enlargement of the heart is situated at the opening of the aorta, the increased energy of the ventricle is exerted to overcome the resistance, and not to communicate a strong impulse to the carotid arteries. On the contrary, when the aortic opening is free, the blood is thrown with increased force upon the brain.

We are surprised to find that M. Rodrigues scarcely alludes to the state of the blood as a probable cause of the disease : he dismisses the question by merely stating that little is known about the blood. Modern researches have, however, proved that a morbid condition of the blood is all-powerful in causing disease. The interesting cases recently published in the "Lancet" by Dr. Bence Jones, have shown that preternatural quantities of the earthy and alkaline phosphates are voided in the urine of patients suffering from cerebral diseases, and Dr. Hitchman, in his Lectures on the Pathology of the Insane, published in the same journal, has mentioned an interesting case of mania which was apparently caused, almost instantaneously, by the introduction of some poison into the circulating fluid. The deteriorated state of the blood in Bright's disease of the kidneys, may easily be supposed to have an influence in producing the particular state of the brain which precedes general paralysis.

M. Broussais, whose fertile brain has detected a gastro-enterite in nearly every disease, is himself quite convinced that an inflamed stomach will cause arachnitis and insanity. There cannot be a doubt that a predisposition to disease of the brain may be kept up by its sympathy with some other organ in the body, either functionally or organically deranged.

Scrofula, again, a fertile source of insanity, must be mentioned amongst the predisposing causes of paralysis.

The exciting causes of the disease are numerous. We may enumerate, strong impressions deranging the impulses and affections, such as losses in business, disappointed ambition, anxiety, grief, &c., also injuries of the head. Many of the predisposing causes may be considered as exciting when they are sufficiently powerful and long continued, which is often the case.

The earliest symptoms of this malady claim our closest attention. On this point we cannot too strongly insist. The disease being fatal in nearly every instance, it becomes of the greatest consequence to detect it at the onset, not only with the view of administering our remedies at a period when we have the best chance of success, but also that we may be enabled to give a correct prognosis at a time when the friends of a patient, from his comparatively healthy appearance, are led to indulge a delusive hope of recovery. As the majority of medical men are not aware of the existence of this disease, and have had little opportunity of recognising it, and as, moreover, they might be induced to consider, with the friends of a patient, that the danger was not imminent, from his joyous manner and the trifling symptoms at the commencement of the disease, we purpose dwelling at some length on the premonitory symptoms.

M. Rodrigues gives a minute detail of the first, almost imperceptible and variable signs of the malady: he also gives us some peculiar views of his own, which we shall presently discuss. He adopts Calmeil's division of the symptoms into three periods; it is a convenient arrangement, but it must by no means be considered absolute in nature. Our attention will be more particularly directed to the first period.

Hesitation of speech and impeded motion of the tongue are the first signs; the patient speaks with difficulty, and stops between each syllable. Some words he cannot articulate; he passes over some lightly and lingers on others. Some patients repeat the same words several times, either at the beginning, middle, or end of the sentence. When asked to show his tongue, he protrudes it straight forwards, and it trembles as if between two antagonizing forces. The mouth is not drawn to either side, but when he is about to eat or speak, there is a slight trembling about the corners of the mouth as if the muscles were hesitating; but this symptom is very transient. The muscles of the face have free play, yet the medical man accustomed to see these cases notices an incipient immobility in the features.

Good and bad symptoms may alternate for some time, and give rise to hopes of recovery which the practised physician knows to be fallacious. The first faint traces of immobility cannot be expressed by language; it is only by the great study of nature it can even be recognised. No paralysis of the face is noticeable, yet, nevertheless, there is an appearance of want of vitality in the features which gives a slightly fatuitous and expressionless look. As the difficulty of pronunciation increases, the immobility of the features becomes more characteristic; the upper eyelid is raised with difficulty; the look is stupid; the cheeks pendulous; and the lips touch, without the power of resting one on the other.

The next organs paralysed are the arms, according to M. Rodrigues. M. Calmeil, on the contrary, states that the legs are first affected, and that he is surprised to see the power which remains in the upper extremities, when the lower limbs are tottering under the weight of the body. M. Lallemand is of opinion that the disease affects the upper limbs in the first instance: he says, that the general incomplete paralysis of the insane appears to begin in the lower limbs, because its progress is so slow, and because the weakening of the lower limbs is more perceptible on account of the strength necessary for the exercise of their functions. He says he has met with patients, termed paraplegic, because they could not walk or sit up; but who, when placed on their backs, were able to raise their legs very high, and exercise them in various ways; but when a glass full of liquid was placed in their hands they generally spilt a portion of it in carrying it to their mouths. In the cases which we have ourselves witnessed, it has appeared that the arms and legs were simultaneously affected. We should, however, be inclined to agree theoretically with M. Rodrigues in attributing to general paralysis a "descending march," as he terms it. It is a well known fact that in cases of hemiplegia, when the patients are recovering, it is the leg, in nine cases out of ten, which recovers first and most rapidly; and it is also true that when one extremity only is affected, in nineteen cases out of twenty, it is the arm only that is paralysed. We may, therefore, naturally conclude that this rule holds good in every form of cerebral paralysis. Mr. Herbert Mayo, who published his views of this peculiarity in hemiplegia in the *Medical Gazette* several years since, attributes the phenomenon to what he calls a palsy stroke, which affects the arms first, on account of their proximity to the brain.

Leaving this question for the present, we will proceed with the description of the symptoms of general paralysis. It is soon discovered that a patient labouring under this disease is unable to support a weight in his hand, or raise his arms above his head. The legs are next implicated; the patient walks a few steps, and knocks the earth as if to support himself, and hesitates when he attempts to walk. Some can walk firmly in a circle, but cannot walk in a straight line; many feel a stiffness in their hams which gives them a peculiar gait; others, again, assume an imposing attitude, and affect a grave and slow carriage, but the muscles do not obey their will. Subsequently, the venous circulation is carried on with difficulty in the inferior parts of the body; the sub-cutaneous vessels become distended, and the skin looks unhealthy.

The premonitory symptoms of this melancholy disease are sometimes intermittent; they disappear for several hours or days, to return with fresh intensity. The loss of motive power is generally less perceptible in the morning and after meals; the walk is then firmer, and the speech more articulate. The paralysis proceeds downwards on both sides, but sometimes attacks one side before the other. The sphincters are not at first affected, although they subsequently become involved in the general paralysis. The muscles of the neck, back, and chest remain free throughout this stage of the disease; still a slight difficulty in breathing and some degree of constipation may be observed; the appetite and digestion are generally good; the temperature of the skin is scarcely increased; the pulse is very little affected; and, strange to say, the patients generally get fat at this period of the complaint.

The second period need not detain us long. It is marked by an increase of intensity in the symptoms, which are frequently aggravated by attacks of congestion. There are sometimes moments of excitement, when the mind is greatly disturbed, and the patient is quite delirious and furious on being contradicted; the face becomes red, the eyes prominent, and the breathing hurried; this state often ushers in an attack of epilepsy. After these paroxysms, the symptoms are discovered to be greatly aggravated, although, during the excitement, the patient has had a temporary increase of muscular power. As the disease progresses, the deglutition becomes difficult, and the patient is apt to be suffocated by the food lodging opposite the larynx. The paralysis extends successively to the muscles of the jaw, neck, abdomen, alimentary canal, and the sphincters; the retina becomes partly insensible to light, and the sensibility of the auditory nerve is blunted.

It is a remarkable fact, that up to this point, the faculty of common sensation remains intact. Dr. Watson, in his valuable Lectures on Hemiplegia, has noticed a similar phenomenon: he says—" The function of sensation (wherefore, I cannot tell) is less frequently abolished or perverted than voluntary motion." M. Rodrigues gives us an ingenious solution of the problem : he says—" The remarkable fact that voluntary motion, and the senses of sight, hearing, and taste, are blunted before the sense of touch, is perhaps explicable by taking into consideration the greater surface of the skin, the numerous and varied points of the cerebro-spinal axis, towards which the nerves abut, and the greater simplicity of this sense." He argues, moreover, that the precise seat of a lesion of the cerebral pulp has more influence over the senses of sight, hearing, smelling, and taste, than it has on the skin, because of the limitation of the origin of the former senses. When the paralysis is incomplete, it bears, he says, much more on motion than on sensation; and that this inequality may be observed, whatever may be the seat of the alteration—that it enters into a law, which holds for all cerebral affections. Sensation and motion do not depend, in his opinion, on distinctive organs, the loss of sensibility being merely indicative of a more advanced state of the disease, and serving as a sign of the extent of the lesion. He goes on to argue, that sensation is merely a passive act of the brain, but that this organ has to re-act when the more difficult and complicated voluntary motion is to be executed. The material alteration in the brain must be great, indeed, he says, " when so elementary a movement as sensation is abolished." This theory is certainly ingenious and simple; but M. Rodrigues does not seem to be aware, that although sensation and voluntary motion do not depend on separate organs, it is rendered extremely probable, by the researches of modern physiologists, that they do depend on distinct *parts* of the nervous system. In the general paralysis of the insane, we conceive it highly probable that the loss of motion is owing to a lesion of those portions of the brain which are connected with volition; and that at the onset of the disease, the parts of the nervous system associated with emotional movement, the consciousness of impressions on the senses, and the sensory and motive tract, are not implicated in the affection. That emotional movement is not seriously disordered at first, is shown by the fact, that the paralytic insane will occasionally have paroxysms of emotional excitement, when he will run wildly about, under the influence of some moral feeling.

The reflex function of the spinal cord (which has been so beautifully brought to light by the investigations of Dr. M. Hall, and shown to be a distinct centre, independent of the brain) remains entire throughout nearly the whole progress of the disease. From the imperfect view which M. Rodrigues takes of the nervous system, we are inclined to think that Dr. M. Hall's researches have not generally met with that attention on the continent to which their merits so deservedly entitle them.

M. Rodrigues' opinion, however, that the function of sensation is simpler than that of motion, is probably correct. The functions of the nerves of organic life may be even yet more simple; for we find that they continue their operations up to the very last period of general paralysis.

In the third period, the general paralysis has reached its height; the patient is in a pitiable state, being far advanced in dementia. Hearing and smell are gone; he may utter a few vague sounds, but speech is lost. The mouth is opened instinctively when food is offered, but deglutition is almost gone, and it becomes necessary to put the aliment into the pharynx. The head is drawn forwards, the lower jaw acts slowly, and the tongue rests at the bottom of the mouth. The legs are now unable to support the body, and the arms lie motionless by the side. Respiration is short, and performed with difficulty, while sensation is nearly gone. There may be constipation, but diarrhœa is most common. Emaciation rapidly ensues, and death closes the melancholy scene. Sloughing bed-sores and gangrene of the lungs are common towards the termination of the disease. M. Guislain attributes the tendency to gangrene of the lungs to imperfect nutrition, on account of the patient's repugnance to food; M. Foville, to the absorption of putrid matter from the sloughing wounds; and M. Rodriques, to the weakened state of the nervous system. No doubt each of these causes may have a share in producing the phenomena.

It will be interesting to notice the character of the insanity associated with general paralysis. Ambitious or exalted monomania is the form of insanity most commonly associated with general paralysis; and in general, it is some mental eccentricity which has first attracted the attention of the patient's friends in these cases, his bodily infirmity having been quite overlooked. The disease, according to M. Bayle, commences with more or less excitement. The patients speak of their fortune and grandeur; that they are able to make considerable purchases, and build palaces. Everything is transformed into gold and silver; and the very pebbles are precious gems, which they hoard with care. Some consider the asylum as a magnificent palace; the persons around them are only there to wait on them; and the strangers who come to visit the institution are regarded as petitioners for their powerful influence. Others consider their detention as a shameful injustice, opposed to all laws, both human and divine. They promise thousands to the physician for their release, and they seek to corrupt the keepers by signing bills for enormous sums of money. One fancies he is a king or an emperor, and exacts homage; another imagines himself to be a Deity, requiring adoration. In an asylum, containing patients suffering from general paralysis, we find barons, peers, generals, physicians, astronomers, poets,—men who know everything, even to the secrets of Providence, and whose power is such as to give motion to the universe. Amongst the cases to which M. Rodrigues refers, one believes himself an absolute prince, and promises honours to his faithful servants, and threatens to exterminate the wicked; another is anxious to distribute millions of guineas; while a third considers himself the son of Jupiter, and visits his father in a balloon.

In the second period, the patients have the same ideas of grandeur, but the insanity is more general. M. Bayle does not admit that the ambitious characteristic is present at this period; but says the second stage is merely distinguished by the greater intensity of the paralysis. M. Rodrigues thinks differently; believing that even in the third period ideas of riches still exist, though the expression is obscure, the patient muttering such words as million, gold, riches, God, in reply to all questions addressed to him. Without doubt, paralysis, he says, may be complicated with all the varieties of mental alienation, and that ambitious monomania may also exist without general paralysis.

Our author is of opinion that general paralysis may be developed after and during mania or monomania, or with dementia. The greater part of his cases appear to have been preceded by insanity. He thinks that the paralysis may precede the insanity; but his evidence for coming to this conclusion is not very fortunate: it is merely the assertion of patients' relatives, who have affirmed that a tottering walk and imperfect speech have preceded the mental alienation. M. Rodrigues thinks that ambitious monomania is not the form of insanity which invariably accompanies general paralysis, although the most common, the insane aspect varying very much in different individuals, and in the same person at different times. The disease generally begins with mania or monomania, and ends in dementia. M. Rodrigues does not agree with Broussais, that the insanity always terminates in dementia, and that paralysis is merely a complication which shortens life. He says he has seen patients live thirty years with general paralysis, remaining all the time maniacal; and has observed others who have fallen into a state of dementia without ever becoming paralytic.

The *duration* of general paralysis is not easily determined, as it appears contingent on the progress of the cerebral disease. Calmeil fixes the second period at an average of thirteen months. Bayle says it varies from two months to six, eight, ten, or even twelve years; and that its mean duration is from one year to a year and a half. Out of one hundred and fifty-nine patients, he found that seventy-three did not live longer than a year.

With regard to *prognosis*, nearly all writers consider the disease incurable. Royer Collard has never seen a perfect cure effected, during a practice of twenty years in a large hospital. Esquirol does not hesitate to say that it is incurable, even in the first period. M. Rodrigues takes a more favourable view : he says it may be cured, but that the cure takes place slowly. When a patient is about to recover, the agitation is first noticed to become less, and the paralysis begins to disappear in the legs, before the arms show any signs of amendment. The paralysis of the tongue is more obstinate, some never recovering perfect speech. Neither is the cure always definitive, as relapses are to be feared; and when they do occur, the patient falls rapidly into the first stage, and the periods succeed one another with more rapidity than before.

In the second part of his work, M. Rodrigues presents us with some interesting cases of partial and perfect recovery, quoted from different authors.

Of nine cases of recovery or amendment cited by M. Rodrigues, one recovered partially as to the insanity, the paralysis remaining stationary; three were perfectly cured; one recovered so perfectly, that at the end of six months a slight impediment of speech alone remained; one remained well for nine months, and then committed suicide; two recovered perfectly as to the paralysis, and partially with regard to the insanity; the other case occurred in an epileptic patient, who recovered perfectly after two relapses, and remained so at the end of three years without the recurrence of any epileptic fit. On the whole, we cannot consider this list very encouraging.

Patients seldom die during the first period, except from attacks of cerebral congestion. In the second period, death is apt to occur from epileptiform attacks and asphyxia. In the third period, the patient generally sinks from general weakness, cerebral congestion, disease of the lungs, or asphyxia.

Complications with general paralysis often remain latent, because the diseased brain is unable to re-act on the sensations which recur to it. In these cases, the paralysis generally runs its usual chronic course; but sometimes it suddenly becomes mature from effusion. Apoplexy is not an uncommon complication. M. Rodrigues mentions an interesting case, in which the patient recovered from two apoplectic attacks and a partial paralysis whilst affected with general paralysis, but who died from the effects of a third attack. This case presented the following interesting physiological fact:—Whilst being bled for one of the apoplectic attacks, the speech suddenly returned during the flow of blood, but on closing the aperture in the vein, the power of articulating as suddenly ceased; on re-opening the orifice, the speech again returned. This experiment was tried several times with the same result. The instantaneous restoration of speech on opening the vein renders it probable that sanguineous apoplexy depends rather on stagnation than congestion, and tends somewhat to support the views of the Scotch physiologists in opposition to those of Dr. Burrows.

Our author not only considers that general paralysis will run its course independently of bodily complications, but also of the associated mental affections. He cites a case of M. Ferrus', in which the general paralysis remained stationary, whilst the insanity became ameliorated. This case, however, cannot be considered conclusive, as the insanity was only subdued, not eradicated. When general paralysis is complicated with paraplegia, he says there is commonly some disease of the spinal cord, or its sheath; or else some effusion of serum, or blood, into the vertebral canal. Phthisis is a common complication in this as in other forms of insanity. The paralytic insane, it appears, are not exempt from ague, cholera, or scurvy.

M. Rodrigues gives us a description of the *pathological appearances* observed in twenty-one dissections. It is unfortunate for science, that when post-mortem examinations are obtainable in subjects who have died insane, the cases are generally of long standing. As usual, we find that those of M. Rodrigues were all of a very chronic character, and were likewise, in several instances, complicated with other diseases. Eleven of the patients were far advanced in dementia, five had suffered from epilepsy, two from apoplexy, two were associated with idiocy, and one with extensive scrofulous disease. In examining cases like these, it will often be extremely difficult to determine what relation a particular lesion may have to the malady in question. The pathology of the brain, in connexion with insanity, is an intricately tangled web, which has disheartened some of the most zealous and able inquirers. Nevertheless, their exertions have not been altogether in vain, and we hope that continued observations may ultimately light upon some "glaring instance," to use a happy expression of Lord Bacon's, which will clear up many of our doubts.

The chief difficulty in the pursuit of pathological truth is to ascertain whether the appearances are causes or effects of the symptoms during life; and should they be generally proved to be merely effects, we must be content to reason from them inductively, trusting that patient observation will ultimately aid medicine as much as it has aided other sciences.

We shall now proceed to give, in the first place, a detail of the morbid appearances found in the above cases, and afterwards to select, for especial notice, those points which appear to be of practical importance; and it is some encouragement to find that many of the organic changes are tolerably uniform in their occurrence.

In one case, the scalp was gorged with blood. The cranial bones were also injected. In two cases, the skull was thin, and presented a milky-white appearance; and in one the bones were eburnated and thickened. In three instances, the walls of the skull were irregular both at the top and bottom; depressions were also met with on the internal face of the skull. In a woman who had suffered from syphilis and the abuse of mercury, the bones of the head were affected with caries and exostoses. Obliteration of the pituitary fossæ and anfractuosities of the brain occurred in one case.

The dura mater was injected, thick, dense, opaque, and adherent in three instances; sometimes it was cartilaginous and osseous, and occasionally perforated, not uniformly, but as if from stretching of the fibres; sometimes attached by its internal face to tumours more or less voluminous, which compressed the contained organ. Very commonly the dura mater lay in folds in front, its cavity being too large for the atrophied brain. The pia mater and arachnoid were injected in various degrees. Distention of the vessels of the pia mater is one of the most common features; the pia mater was sometimes pale and greyish. The arachnoid was thickened and opaque in nearly every instance, but not invariably throughout its whole extent; most commonly in that part which covers the superior and anterior portions of the brain, especially on either side of the superior longitudinal sinus, and on its internal face. Granulations on the arachnoid membrane were found in several instances; and in one case, the lining membrane of the ventricles was so studded with these minute prominences as to feel like fine sand. The granulations were most frequently met with in the ventricles, and the inferior occipital fossa.

Serous infiltration of the cellular membrane of the pia mater, and of the tissue which separates it from the arachnoid, was frequently found, especially in the anfractuosities. In the greater number of cases, serum was found in the cavity of the arachnoid membrane; this serum was either transparent, greenish, or bloody, and of varying consistency. In one instance, the pia mater appeared to be remarkably dry; the ventricles were more or less full of serum, and frequently the distention was so great as to destroy the middle wall. In a case of M. Bayle's, the pressure of the liquid had occasioned a rupture of the arachnoid membrane which lines the inferior wall; the serum had escaped into the tissue of the pia mater which lines the middle lobes of the brain, and had formed under each lobe a kind of irregular pouch, transparent and soft, and lodged in the lateral and middle fossa of the base of the cranium. On inclining the bodies, serum frequently flowed from the vertebral canal.

Effusion of blood between the layers of the arachnoid rarely took place in both hemispheres, but it was found that in flowing towards the base of the brain it produced the same symptoms as if it occurred in both. Calmeil gives a case of extensive effusion of blood of a violet colour, covering the upper and lateral parts of this right hemisphere, penetrating even to the base of the cranium, and bathing and compressing the peduncles of the brain and optic thalami. In one case cited by M. Rodrigues, there was an effusion between the dura mater and the external layer of the arachnoid membrane, which had completely dissected that membrane from the great falx to the tempero-parietal suture, and from the coronal fossa to the posterior edges of the parietal bones on each side. There was sometimes found in the cavity of the arachnoid membrane an albuminous effusion, which had some analogy to trembling jelly, and which was easily removed; it may occur entirely at the base of the brain, but it is occasionally met with between the arachnoid membrane and pia mater.

False membranes are frequently found, resting generally on the convexity of the hemispheres, and on the anterior part on one or both sides. In M. Calmeil's cases these membranes were partly fibro-cartilaginous. M. Rodrigues thinks that suppurative meningitis may sometimes produce general paralysis. The pus, in these cases, he says, is situated between the fold of the arachnoid and the pia mater, and presents a different form from ordinary pus, having a creamy-whitish appearance, and being composed, according to Magendie, of irregularly-formed globules, less than those of ordinary pus, but larger than those of blood, being, as it were, in a state of transition between the two.

Hyperæmia of the brain was met with in different stages; the cerebral pulp was red, injected, and slightly tumefied, and when sliced, small drops of blood oozed from the cut surface.

Softening of the brain assumes a variety of forms; it either penetrated to some depth, or extended over the whole cerebral surface; it attacked some parts only, or it occupied the whole cerebral mass; sometimes there was a simple diminution of consistency; at other times, the structure was completely diffluent. In one case, the face of each posterior lobe, the cornua ammomis, the edge of the middle lobes, the anterior part of the cerebellum, and the tuber annulare, were all in a state of putrefaction. Softening may take place without change of colour; in the grey substance it was found rosy, red, or a deep brown; in the medullary portion of the brain it presented a pale hue, sometimes greenish or yellow.

Hardening of the brain occurred as frequently as softening, and was accompanied by either hyperæmia or anæmia. In some cases, the cortical substance and the ventricular walls were indurated; the other parts, nevertheless, being in a normal state. In one case, softening of the superficial parts of the brain occurred whilst the deep-seated portions were hardened. In one case, cited from Calmeil's work, in which the skull had sustained a fracture, with loss of substance of the parietal bone, the part of the convolutions contiguous to the bony aperture was firm and resisting, and covered a layer of hardened brain. In another instance, the colour of the hardened brain varied from a light coffee colour on the outside of the brain, to a reddish tint on the inside. The cerebellum and pons varolii were indurated in one case.

Other lesions are to be met with in general paralysis; M. Leuret notices two superficial erosions on the middle lobe of the right side. M. Rodrigues says he has met with ulceration of the inferior face of the cerebellum throughout the whole extent of the vermicular eminence; he says he has also found tumours on the cerebral hemispheres, on the corpus callosum, at the basis of the cranium, and throughout the whole depth of the brain, causing prominences in the ventricles; and in one case of general paralysis, he discovered a tumour compressing the spinal cord, and reducing it to the thickness of a ribbon. He has also occasionally seen fibre, cartilaginous and bony tumours, hydatids, tubercle, cancer, and growths on the pia mater, in the bodies of those who have been afflicted with general paralysis.

M. Rodrigues has detailed two interesting cases of œdema of the brain, which he cites from M. Ferrus' collection of cases. One of these patients was affected with general paralysis, combined with dementia and epilepsy.

Having thus briefly enumerated all the signs which the morbid anatomy of general paralysis reveals to us, we shall, in the next place, offer a few observations on some of those points which appear to be of practical importance.

The condition of the skull first demands our attention. Writers on insanity have generally noticed either a defective form or want of symmetry in the heads of the insane. One English writer, however, of high anthority, has stated it as his opinion, that the skulls are generally well developed in those afflicted with this peculiar malady; this view of the case does not accord with M. Rodrigues' observations, who has noticed an irregular form of the cranium in several instances; and in all the cases which we have lately seen, the skulls were strikingly ill-formed. In some of the patients, there was a remarkable depression of one side of the frontal bone, as if flattened by a mallet; in one man, the head was singularly round, like that of a woman. M. Rodrigues informs us, however, that Bichet, whose genius was so powerful, and who advanced the opinion that a want of symmetry in the two sides of the head was an obstacle to the development of intellect, had himself an irregularly-shaped head; thus offering, in his own person, a singular contradiction of his views.

Injection of the vessels of the pia mater, and thickening of the arachnoid, combined with effusion, are found in the greater number of cases. M. Magendie has advanced an opinion that the arachnoid does not resemble other serous membranes, inasmuch as the liquid, in cases of effusion on the brain, is found between it and the pia mater, whereas, in affections of the pleura and peritoneum, the serum is always found in their cavities. M. Rodrigues is, however, perfectly convinced that, in cases of chronic meningitis, he has generally found a variable quantity of liquid in the arachnoid cavity as well as in the subserous space. The frequent occurrence of serous effusion in cases of general paralysis led Messrs. Bayle and Esquirol to ascribe the disease to meningitis. They were hasty, however, in drawing this inference, as effusion of serum is very commonly found in cases of insanity, uncomplicated with general paralysis.

It has been remarked by authors, that hardening of the brain in general paralysis is often accompanied by diminution of volume. As the dura mater cannot, therefore, adapt itself to the brain in these instances, and as the serum, though exhaled in great abundance, is not sufficient to fill the vacuum, the dura mater naturally falls into folds, in a transverse manner. M. Rodrigues suggests that this may be a cadaveric effect, the serum having passed into the neighbouring tissues; he also thinks that the atrophy and hardening of the brain in old age may be owing to a deficiency of water in its substance; that the fluid of the brain varies with age; and that it may even diminish to so great an extent as to leave the nervous pulp completely dry; and, moreover, that it may, on the other hand, accumulate in such abundance as to constitute what is commonly called œdema of the brain. M. Magendie, who has devoted much attention to the subject of liquid effusion in the brain and the vertebral canal, considers it as very probable that the ventricular exhalations take place at the expense of the brain, which furnishes the fluid for the purpose; and to this cause he attributes certain cases of internal hydrocephalus, accompanied by a closing of the entrance of the encephalic cavities.

With regard to œdema of the brain, M. Scipio Pinel considers it to be merely an accidental production from cerebral irritation, and occurring in individuals predisposed to anasarca. M. Rodrigues and Dr. Etoc Demary differ from Andral, who thinks that œdema of the brain produces no particular symptom; they, on the contrary, consider that it must compress the cerebral tissue, and produce functional disturbance.

It occurs to us that numerous errors and conflicting opinions have arisen in cases of effusion on the brain, in consequence of writers not taking into consideration the question as to whether the fluid had been suddenly or gradually effused. We all know that a very trifling extravasation of blood into the corpus striatum will cause death when suddenly forced upon its structure. It is also generally well known that the greater part of one hemisphere of the brain may be completely disorganized for some time before death, provided the changes have been gradually brought about; and still more strikingly we have all observed, no doubt, the extraordinary unfolding and extension of the fibres of the brain in chronic hydrocephalus, without derangement of the intellect or any material impediment to the corporeal functions. An attentive consideration of these well-known facts would, we think, clear up many apparent discrepancies in cerebral pathology.

Messrs. Calmeil and Lelut believe that the atrophy, in protracted cases of general paralysis, may have been occasioned by effusion of serum; M. Rodrigues doubts the possibility of such an effect; but if we keep before us the distinction alluded to above, we can easily conceive, that after a long period (and these lesions invariably occurred in protracted cases) the brain might have gradually given way to the incessant, the imperceptible encroachment of the liquid effusion.

The cause of altered consistency of the brain is a "vexata questio" which next demands our attention. Induration and softening of the brain, we perceive, are common occurrences in chronic cases of general paralysis, and it is extremely difficult in these cases to determine the nature of the vital changes which have induced the lesions. M. Rodrigues, in a hasty and dogmatical manner, asserts that all alterations produced by inflammation begin with softening, and end with hardening, but that it is not correct to infer that every kind of induration indicates previous inflammation. He also states it as his opinion that induration may be produced by an operation like that which presides over the hypertrophy of organic tissues in general; and that, in consequence, the external surface of the brain being softened and the membranes inflamed, the tissues immediately adjacent must be subjected to increased excitement, under the influence of which, the circulation becomes accelerated, and the nutritious particles accumulated, and that these particles, from the unyielding nature of the skull, must necessarily be pressed on each other, and hypertrophy without increase of volume be the result. This sweeping conclusion is opposed to the general laws of the animal economy; for we find that the ordinary results of inflammation, ulceration, suppuration, mortification, &c., are alterations diametrically opposed to his statement, that hardening is the end of all inflammatory disorganizations. We all know that induration may be an occasional effect of inflammation, but not in the singular manner which he supposes. Indeed, his opinion, that an inflammation on the surface of the brain is capable of exciting that action in the substance of the organ which leads to increased nutrition, is too fanciful to be entertained.

Induration is no doubt the effect of various causes. The hardening of some structures, as age advances, is a natural result which must not be overlooked in pathological examinations. Sometimes a tissue is hardened in consequence of an altered condition of the fluid exhaled into its cells, showing that the disease may be occasioned by an altered state of the blood. Induration is also, in many cases, preceded by an inflammatory condition of the cellular membrane, as commonly seen in affections of the *lung*. It does not, however, necessarily happen that because an irritation has produced an induration, that it should continue; for we frequently find in an inflamed brain, portions of hardened pulp, which look like inorganic matter, imbedded in the cerebral pulp. Sometimes, according to Andral, a secondary irritation will ensue, and restore the indurated part to its healthy condition. M. Lallemand has supposed that partial induration of the brain may be a mode of recovery from softening, and there are certainly some general pathological facts which appear to support his view.

M. Rodrigues recognises three species of induration of the brain in general paralysis—hypertrophic hardening, from irritating causes; inflammatory hardening, or that which is the result of inflammation; and atrophic hardening, which has been occasioned by serum compressing the brain. The first and last species, he believes, affects generally the whole cerebral mass; the second partially only, but marked by a change of colour.

Dr. Delaye, who discovered hardening of the brain in the greater number of his cases, attributes great importance to induration of the cerebral mass, and considers it to be a frequent cause of paralysis in the insane, and he gives, in his inaugural thesis, several facts which favour this opinion. Foville also considers that hardening of the medullary structure of the brain will cause general paralysis. M. Rodrigues thinks that it may have some share in producing the paralysis, but very justly observes, that its degree of importance is certainly problematical, inasmuch as it is never found alone, other lesions, sufficient to cause the disease, being generally noticed at the same time.

Softening of the brain is, generally speaking, the effect of inflammation; and occurs, as we have previously noticed, very commonly in general paralysis. M. Rodrigues is naturally at a loss to know whether the softening took place at the commencement of the paralytic symptoms, or towards the termination of the disease. Although it appears improbable that a patient could live long with extensive softening of the brain, yet Lallemand and Abercrombie cite examples, which render it probable that patients may live a year, or more, whilst suffering from this affection.

We are surprised to find M. Rodrigues, after the evidence which he has brought forward to the contrary, stating it as his decided opinion that the paralysis of the insane differs from ordinary paralysis solely in the circumstance of its progress being more slow. In justice to him we will proceed to give a fair view of his opinions on the nature of the disease, and then state our own reasons for not agreeing with him in several of his conclusions. He commences by saying that the symptoms of the paralysis do not vary, whether arising from an effusion of serum, or from a chronic alteration of the two hemispheres, or whether a central portion of the brain, the cerebellum or the spinal cord, be affected. The disease, in his opinion, commences with sanguineous congestion, which is discoverable by symptoms differing in intensity, and which may be occasioned by a blow or some other cause. This congestion, he says, gives rise to chronic inflammation of the membranes and cerebral disturbance, indicated by delirium and agitation; the inflamed arachnoid, in the next place, pours forth serum upon the surface of the brain, into the ventricles at the base of the brain, and into the vertebral canal, producing general paralysis. If the progress of the complaint is slow, it is because the serum is slowly secreted. He asserts that chronic meningitis is the cause of insanity in the greater number of cases, and that it is probable, therefore, that inflammation of the serous membrane should give rise to a liquid effusion.

After giving this confident opinion, he singularly enough proceeds to state that it frequently happens, on examining bodies, that the brain is found infiltered and the ventricles distended by serum in individuals who, throughout life, had never exhibited any signs of paralysis. Autopsies, he says, frequently reveal that meningitis is connected with inflammation of the surface of the brain; and in these cases the delirium which occurs before death must be attributed to the meningitis; whilst the early symptoms of the disease—viz., excitement and disturbance of the intellect, the headach, the sensibility of the retina, the cutaneous pains, and the contractions of the muscles, are all, he thinks, referable to meningitis. When the brain is conjointly inflamed with its membranes, he says the paralytic symptoms will vary, but he does not admit coma as an effect of encephalitis. M. Rodrigues adduces the following authorities in support of his opinion; the first he mentions is M. Bayle, who has reported fifty cases of general paralysis, in the whole of which he found the signs of chronic meningitis. The Memoirs of the Académie des Sciences for the years 1705 and 1706 contain some facts mentioned by Littre and Geoffroi which tend to support the views of M. Bayle. M. Rodrigues proceeds to cite M. Chiarugi, a physician at Florence, and M. Neumann, physician to the Hôpital de la Charité de Berlin, as authors who have published cases precisely similar to those of M. Bayle's. Professor Rech, he says, has also published some observations on general paralysis, showing that opacity of the arachnoid and effusion of serum were present in all his cases. M. Calmeil's cases and those of M. Ferrus, published in the *Gazette Medicale* and the *Lancette Française*, are also brought forward by M. Rodrigues as corroborative of this opinion. Notwithstanding the evidence which M. Rodrigues has adduced, we consider that the cause of general paralysis is involved in much obscurity, and that the short time which has elapsed since the disease was first recognised has not been sufficient for investigations into the nature of the disease. Enough, however, we think is known to enable us to determine that it is a distinct and specific form of paralysis, having a peculiar origin, progress, and termination. M. Rodrigues has stated that it is not different from ordinary general paralysis; but a very slight comparison of the two diseases will serve to show his mistake.

General paralysis in the insane comes on slowly and insidiously, and commences with paralysis of the muscles of the tongue; ordinary general paralysis, on the contrary, commences as an apoplectic seizure, paralysing instantly all the movements of the body; or else as a spinal disease, in which the muscles that derive their nerves from the brain and medulla oblongata are seldom implicated except towards the termination of the affection; whereas, in the paralysis of the insane, disordered action of the muscles supplied by the lingual and facial nerves are amongst the symptoms first observed.

With regard to the pathological features which he considers as corroborating his opinion that the disease originates in an inflammation of the membranes, we would observe that they were noticed for the most part in protracted and complicated cases, and similar appearances were constantly observed in cases of insanity without paralysis; therefore little reliance can be placed in them. With respect to the symptoms (the delirium, &c.) which he has adduced as signs of meningitis, we all know how little pathognomonic they are of inflammation of the membranes, and that simple irritation of the brain is sufficient to cause them. M. Lallemand has, moreover, mentioned facts which go far to prove that an inflammatory state of the substance of the brain must tend to compress its structure, and thus obliterate rather than excite its functions. Mr. Solly, however, it must be stated, thinks, on the contrary, that inflammation of the cineritious part of the brain stimulates it like alcohol. This difference of opinion may arise from a proper distinction not having been made between the effect produced by the velocity of the current of blood as it passes through the brain, and that caused by hyperæmia; the former must excite, the latter depress its functions. It must also be recollected that general paralysis may leave after death no trace of appreciable alteration of the brain or its membranes; such cases have been mentioned by competent observers. M. Lelut, in the first number of the *Annales Medico-Psycologiques,* for January, 1843, has noticed two cases of general paralysis, combined with dementia, in which no alteration of the brain or its membranes could be discovered after death. Sir B. Brodie has mentioned a very remarkable case of paralysis that commenced in the muscles of the legs, and gradually extended to the other limbs, and which existed during a period of ten years; after death, the most careful examination could not detect any disease, either in the brain or spinal cord. We are, therefore, compelled to confess that we are still on the threshold of our investigations with regard to this singular disease, and that it is to future inquiries we must look for more precise information respecting its nature.

It is probable, if we may hazard a conjecture, that the seat of the disease will be ultimately found in those portions of the brain which preside over the voluntary movements, and which may produce the paralysis by being either sympathetically or directly affected. M. Rodrigues has mentioned two cases which commenced with tremblings of the limbs, somewhat resembling the muscular agitations perceived in delirium tremens; but there is another disease which we think more closely resembles the paralysis of the insane, and the study of which may ultimately serve to throw some light on the subject; we allude to paralysis agitans. Shaking palsy often commences imperceptibly and progresses slowly; it may commence in the head or in the arms, which may remain affected for years; after a while the paralysis extends to the legs, which become weak and tremulous, and unable to obey the will; at a more advanced stage, the power of speaking and eating is lost; the urine and fæces are passed involuntarily; coma at length ensues, and terminates in death. Although the two diseases are distinct, there is, nevertheless, a remarkable coincidence in many of the symptoms. It is also a singular fact that induration of some parts of the nervous system has been discovered occasionally in both diseases. The peculiar effect of lead and arsenic on the movements of the muscles must not be overlooked in our researches. The chemical composition of the brain, a fourth part of which is composed of oleaginous matter, in a state of health, ought also to be investigated, in order to ascertain whether it has undergone any change. In consequence of the general opinion that this malady is incurable, the treatment has been too much neglected. M. Rodrigues complains of the defective manner in which the treatment of diseases in general is discussed by modern writers. This may be the case on the Continent, where the expectant mode is adopted; we, however, do not plead guilty to the charge in England.

In the first period of the disease, when the paralysis is slight, M. Rodrigues recommends the most heroic treatment; he says, that neither advanced age, nor difference of sex, nor apparent weakness of the pulse, contraindicate the use of the lancet. We must use it, moreover, not only once but several times. Although some robust patients may bear frequent venesections, we think it cannot be too frequently enforced that the brain, in chronic, as well as in acute insane cases, is greatly injured by depleting measures. M. Rodrigues has himself mentioned a case in the early part of his work, in which a patient became paralytic, immediately after convulsions, produced by bleeding. M. Pinel was so convinced of the danger of general bleeding in chronic mania, that he was fearful of drawing a drop of blood in patients labouring under insanity, lest they should be hurried into dementia. No doubt M. Pinel carried his views too far, and there is every reason to suppose that in the first period of general paralysis, and in many forms of insanity, local bleeding is often of essential service. When the first appearance of the disease has coincided with the suppression of the catamenia, of hæmorrhoidal or other discharges, the restoration of these secretions is often attended with marked benefit. Both Messrs. Esquirol and Rodrigues mention cases in point.

When the general paralysis occurs in a debilitated state of the body, induced by venereal or other excesses, M. Rodrigues recommends cold baths, light tonics, and aromatics. But even in these cases we are surprised to find he advises an occasional abstraction of blood from the arm, although violent purges and strong stimulant excitants of the nervous system, such as strychnine, he very properly condemns as injurious. Everything tending to cause cerebral excitement, he says, should be withheld; intellectual exertion proscribed; and communications of an unpleasant nature avoided. The douche, which has frequently been boasted of as a powerful agent in those cases, he considers hurtful, in consequence of the reaction which often occurs after its use. He also considers ice, placed on the head, as equally objectionable.

There is a mode of applying cold to the head, recommended by Dr. Arnott, to which he does not allude, but which, we think, deserves attention in all cases requiring the continued application of cold. Dr. Arnott ingeniously suggests that a bladder, with two pipes attached to it, should be placed on the head; by means of these pipes an attendant is to maintain, by pouring water into one pipe and allowing it to flow out of the other, a constant stream of cold water, which will pass over the head of the patient, and keep it incessantly cold, without the possibility of reaction, provided the attendant does not desert his post.

Digitalis, a remedy greatly extolled by Dr. Locher, of Vienna, and which he administered in large doses, was used with advantage in two of M. Rodrigues' cases; and it was tried extensively at Hanwell, but without any apparent benefit.

In a case cited to show the beneficial influence of digitalis in this disease, there was a combination of epilepsy with the general paralysis, a connexion which has been supposed never to occur. And what is still more strange is, that the occurrence of a severe epileptiform attack temporarily rectified the disordered state of the mind. This fact would appear most incomprehensible, had we not seen analogous phenomena with regard to bodily disease. It is not uncommon for patients in hospitals, who have been suffering from indolent ulcers, to be suddenly attacked with erysipelas. In this case we have frequently observed, that after the eruptive disease has passed away, the ulcers have put on a healthy appearance, and quickly healed. It would seem, in these cases, as if the disordered parts were righted by the general commotion of the system.

M. Rodrigues speaks lightly of sedatives and antispasmodics, such as opium and musk. He says no good has ever been derived from their use. Emetics have been tried extensively by M. Royer Collard and M. Bleynie, but were found rather to aggravate the disease. Tartar emetic has been tried by M. Rech, in five patients; the first was maniacal, the second was a case of dementia; the third and fifth were cases of dementia, combined with incomplete general paralysis; and the fourth had ambitious monomania with general paralysis. Of these cases, the maniac was the only one who was benefited by the remedy, whilst in the four other cases the emetic appeared completely useless. When the disease has reached the second period, M. Rodrigues believes general bleeding is not indicated, and that revulsives must then form the principal part of the treatment. If the digestive organs be exempt from inflammation, he recommends laxatives and enemata. It will be necessary, moreover, to watch the digestive organs very attentively, for in consequence of the alterations of the brain, gastro-enteritis, in great intensity, might remain undetected. Revulsives on the skin, setons, moxa, issues, &c., he considers useful, and they were successfully tried by Messrs. Bayle and Royer Collard. M. Esquirol, on the contrary, found the application of moxa unavailing when applied to the top of the head in cases of dementia complicated with paralysis. He also applied the actual cautery to the necks of patients who exhibited signs of paralysis, without any beneficial result. M. Fabret, on the other hand, obtained great success by the application of the cautery, in the case of an insane patient, at the Saltpétriere; and M. Voisin read a paper, at the Académie de Médecine, in which he gives an account of the application of the cautery in ten cases. In only one instance was it found useless, whilst in the nine other cases an improvement, more or less appreciable, took place in the mental disturbance, and in the disordered functions of sensation and motion.

We are surprised to find that M. Rodrigues never alludes to mercury as a remedial measure in this disease, although he considers it to depend on inflammation. Mercury has, nevertheless, been given with advantage for general paralysis in this country, as we shall hereafter show. M. Rodrigues, no doubt, shares the prejudices of many of his countrymen against this mineral. In one case that he details the disease ensued on the suppression of a venereal rash; and although the indications of treatment were clear, we are astonished to find that he never administered a particle of mercury.

In the latter stages of the disease, M. Rodrigues recommends external counter-irritation, whilst internal revulsives must be omitted on account of the diarrhœa, which then becomes very profuse. He mentions that M. Boudin, an old army physician, has much extolled the power of nitrate of silver, either taken as a pill, or in the form of an enema, as a remedy for muco-enteritis, and that M. Ferrus recommends quinine and other tonics in the last stage of the disease. Should convulsions, epileptiform attacks, or apoplexy supervene, M. Rodrigues advises, even in the latter period of the disease, that antiphlogistic measures should be employed, provided an effusion of serum is not supposed to be present; for, should this be the case, he thinks the symptoms would be aggravated by general blood-letting. We, however, consider that general bleeding is to be deprecated in these complications. Dr. Conolly justly observes, that a flushed countenance and an excited manner must not lead us to suppose that blood-letting is necessary; and he mentions two instances in which full bleeding was practised, in consequence of the supervention of violent epileptiform paroxysms, and most serious effects were the result: the patients became reduced to an extreme degree of mental and bodily debility, from which they never rallied.

In the recent reports of the Commissioners in Lunacy, there are numerous data from medical men connected with asylums in this country, relative to the medical treatment of insanity. With regard to the treatment of the malady under consideration, the information afforded is, on the whole, very unsatisfactory and conflicting. The majority of the gentlemen who have sent in their evidence think that little can be done beyond palliative treatment. From a review of these statements, it would appear that general bleeding was had recourse to in none of the cases, but that local bleeding, by cupping or leeching, and counter-irritation, were frequently of service in the outset of the disease, but that in subsequent stages of the complaint they were useless. When the disease is advanced, a generous diet, attention to the secretions and excretions, freedom from excitement, and cleanliness, will often afford the patients a comfortable existence for many years. In some cases, ammonia, porter, and tonics, combined with a generous diet, appear to have the effect of prolonging life. Setons were used with advantage, it seems, in the practice of only one medical man; air or water beds are strongly recommended, in the advanced stages of the disease, when the susceptibility to bed sores is great. The actual cautery was applied to the nape of the neck in one instance without benefit. Friction, warm baths, and alterative doses of mercury are strongly recommended by several practitioners. In some cases, attended with great irritation and distress in consequence of the elimination of phosphatic salts in the urine, opium was found of great service. One physician, on the contrary, found opium of no service in allaying the excitement which often accompanies the disease, but derived the greatest benefit from hyoscyamus. Iodide of potassium, combined with vegetable tonics and a warm temperature, proved beneficial in the practice of another. Creosote and elaterium were also tried without any marked result. Diuretics were of some service when œdema of the feet existed.

The temporary benefit obtained by medicine in this disease is often so striking as to lead medical men to suppose that they have cured cases, when speedy relapses too often show how fallacious have been their conclusions. M. Rodrigues justly observes, that the hygienic cure of the paralytic insane is of the greatest importance, either as curative treatment, or as means of rendering the patients more comfortable, when all hopes of recovery are at an end. If the unfortunate patients are able to walk, they will merely require to be watched; but on their reaching the third period of the disease, they are generally suffering from complete dementia, and then require much more care. They must be fed, or they will die of hunger, and their cleanliness must be especially attended to; the nurses must remove them from their beds every day, and place them in a warm, though airy spot, for several hours. The bed sores, which occur towards the close of the disease, require the closest attention. Many of the horrible wounds produced by the pressure, and which are described in M. Rodrigues' cases, might, we think, have been prevented by a timely use of the hydrostatic bed. He recommends local applications to these sores, as lycopodium powder, sprinkled on lint, soaked in citron-juice; a solution of creosote; tannate of lead; camphor, charcoal, &c. These astringents and antiseptics are no doubt calculated to induce a healthy action of these ulcers.

We shall conclude our notice of this work, by referring to a judicious suggestion of the author with respect to the management of convalescents. He remarks, that generally speaking, the treatment of cases is not sufficiently prolonged; that the physician should resist the entreaties of the patient and his friends to dismiss him until he is completely convinced that the brain may resume its functions without danger. He must, at the same time, recommend the patient not to exercise too severely an organ which, more than any other, requires the most careful management, from the peculiar delicacy of its structure.

After having given M. Rodrigues' opinions such an attentive consideration, we have neither time nor space to enter into his merits as an author. The obscurity and exaggeration of his style we were frequently compelled to deprecate; and occasionally we were startled by the astounding dogmatism of his assertions; nevertheless, he has offered a good digest of all that is known on the Continent on the subject of the general paralysis of the insane. We cannot consider him as a very original writer; but he certainly deserves credit for the method and industry with which he has investigated the various and intricate phenomena of an obscure and peculiar disease.

